# Supplementary material for: Tracking lake drainage events and drained lake basin vegetation dynamics across the Arctic
Source: Nat Commun. 2023 Nov 15;14:7359. doi: 10.1038/s41467-023-43207-0 (PMC10652023; doi:10.1038/s41467-023-43207-0)
Supplement: Supplementary file 1 — Supplementary Information [file 41467_2023_43207_MOESM1_ESM.pdf]

Supplementary Materials for

Tracking lake drainage events and drained lake basin vegetation dynamics across the Arctic

Authors: Yating Chen[1,2,3*], Xiao Cheng[2,4*], Aobo Liu[1,2,3*], Qingfeng Chen[1], Chengxin Wang[1,5]

Affiliations:
[1]College of Geography and Environment, Shandong Normal University; Jinan 250014, China.
[2]Key Laboratory of Comprehensive Observation of Polar Environment (Sun Yat-sen University), Ministry of Education, Zhuhai 519082, China
[3]College of Global Change and Earth System Science, Beijing Normal University; Beijing 100875, China.
[4]School of Geospatial Engineering and Science, Sun Yat-sen University, and Southern Marine Science and Engineering Guangdong Laboratory (Zhuhai), Zhuhai 519082, China
[5]Key Research Institute of Yellow River Civilization and Sustainable Development & Yellow River Civilization by Provincial and Ministerial Co-construction of Collaborative Innovation Center, Henan University, Kaifeng 475001, China.

*Corresponding author. Email: chenyt2016bnu@gmail.com; chengxiao9@mail.sysu.edu.cn; lab2016bnu@foxmail.com

The PDF file includes:

Figs. S1 to S13
Table S1 to S7
Supplementary References

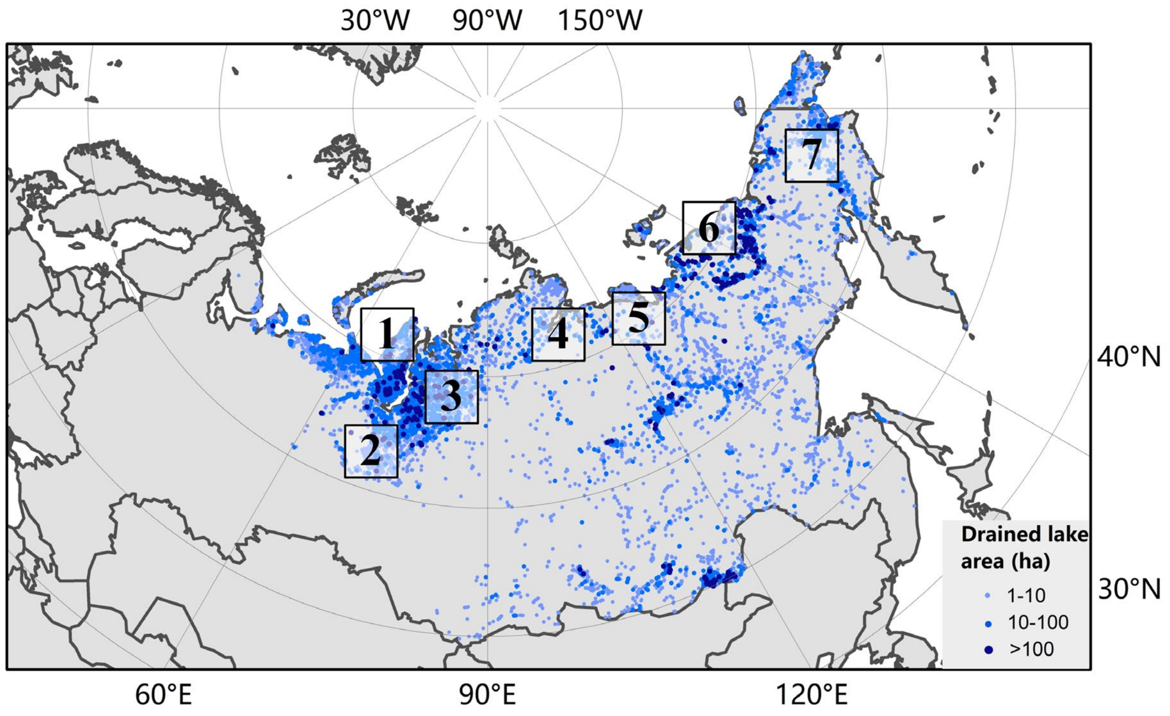

Fig. S1.

Distribution of lake drainage events in the Russian region. The numbered regions marked on the map are: 1. Yamal Peninsula; 2. West Siberian Plain; 3. Gydan Peninsula; 4. Northern Siberian Lowland; 5. Lena Delta; 6. Kolyma/Yana-Indigirka Lowland; 7. Anadyr/Chaun Lowland.

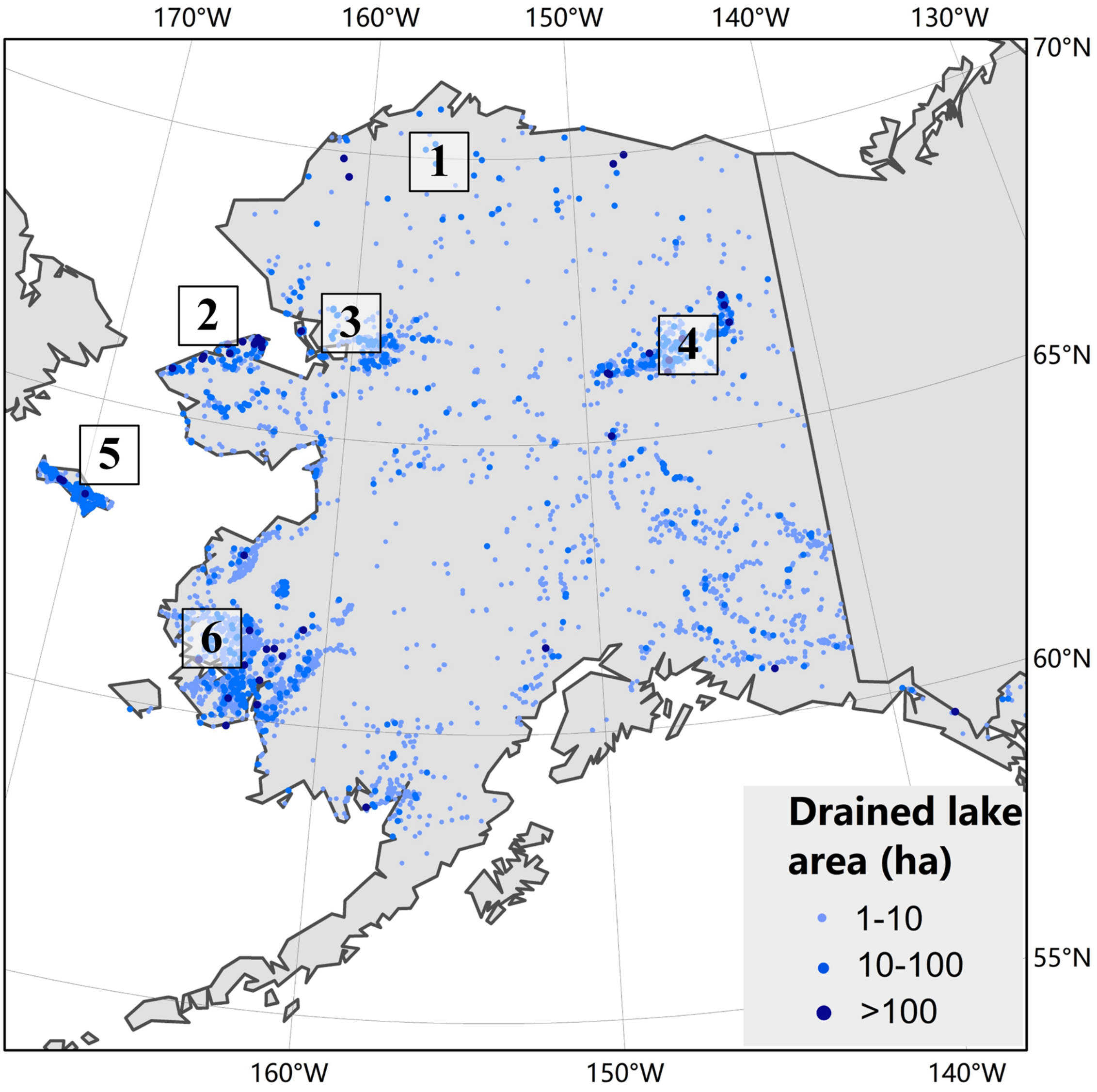

Fig. S2.

Distribution of lake drainage events in the Alaska region. The numbered regions marked on the map are: 1. Alaska North Slope; 2. Northern Seward; 3. Selawik/Kobuk lowlands; 4. Yukon Flats; 5. St. Lawrence Island; 6. Yukon-Kuskokwim Delta.

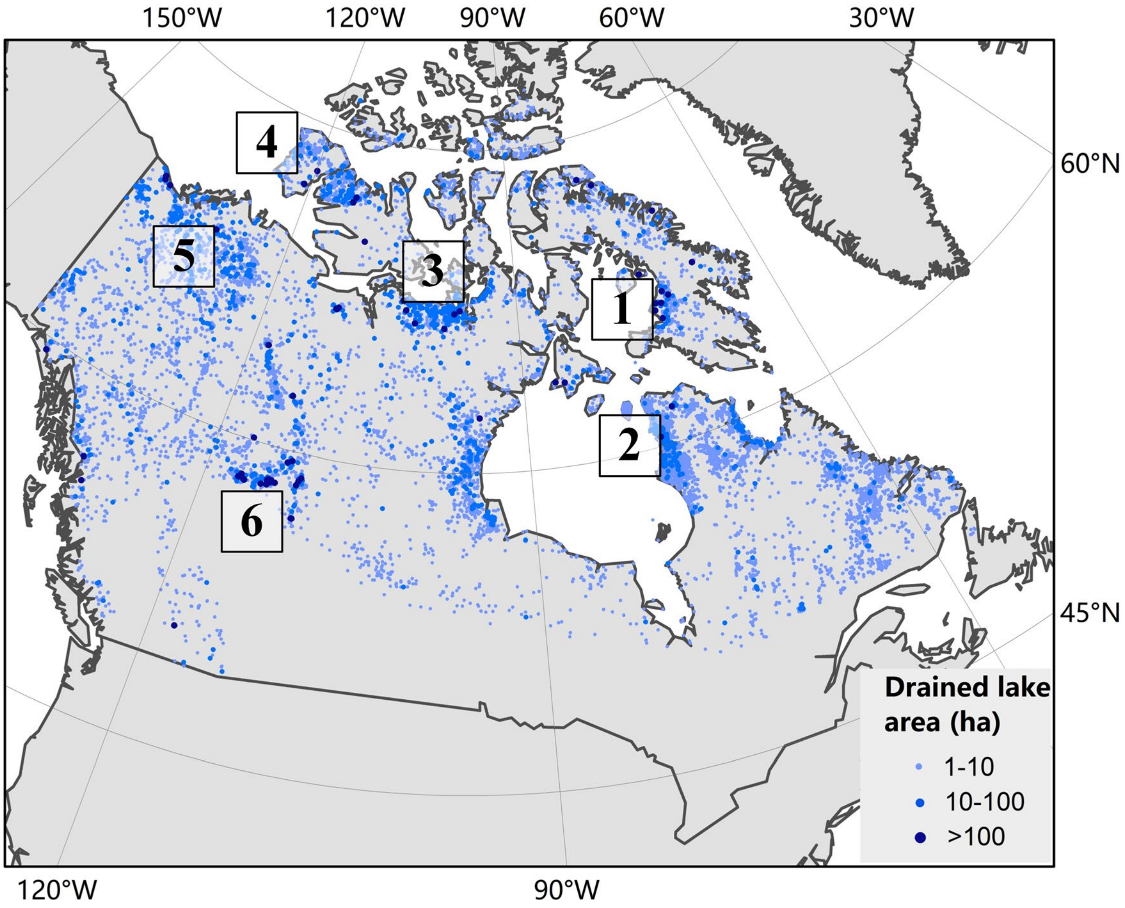

Fig. S3.

Distribution of lake drainage events in the Canadian region. The numbered regions marked on the map are: 1. Koudjouak Plain; 2. Hudson Bay Lowlands; 3. Cambridge Bay Lowlands; 4. Banks Island; 5. Canadian Western Arctic coastal/Taiga plains; 6. Peace River Bank.

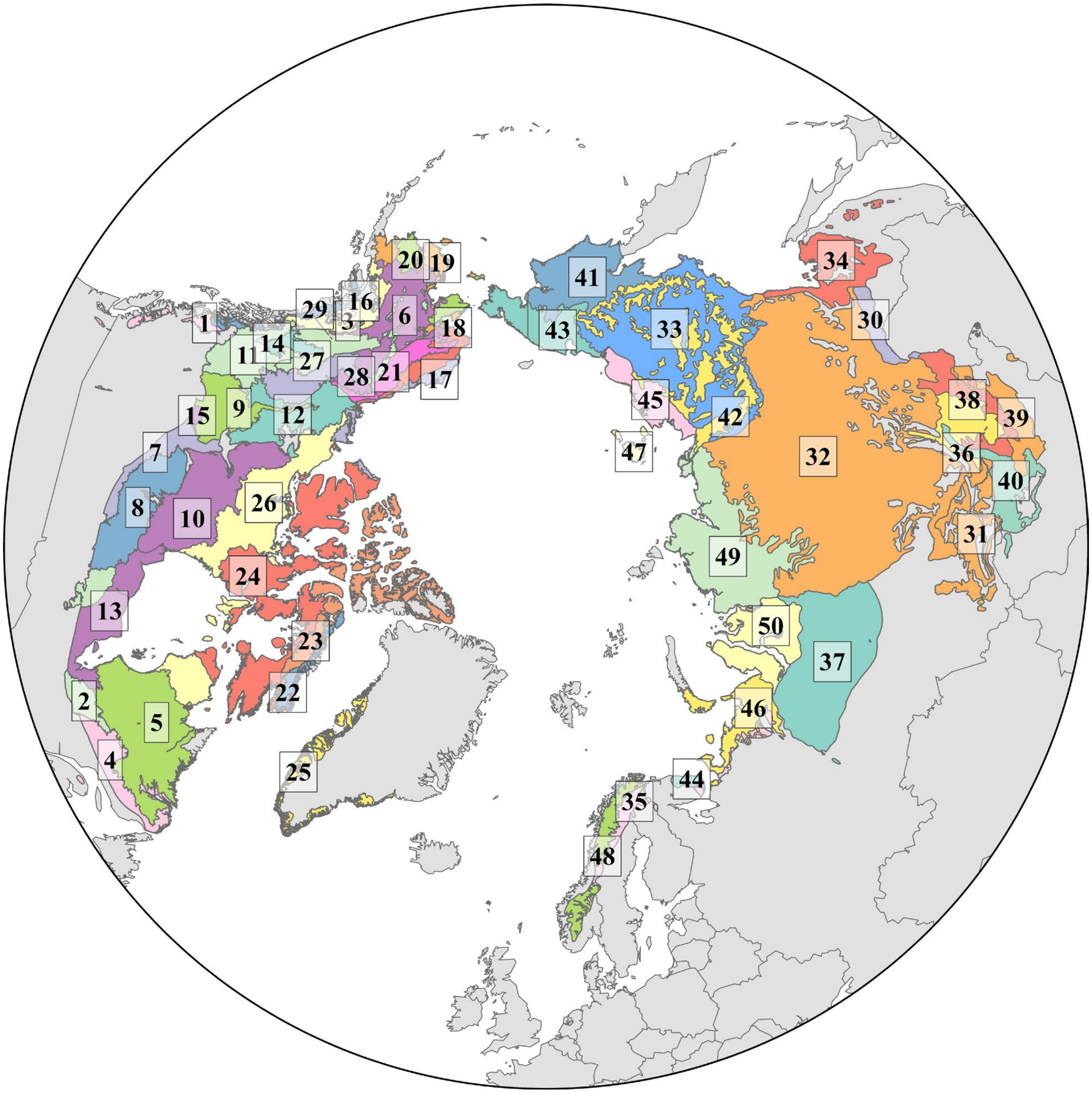

Fig. S4.

Ecoregion delineation in the circum-Arctic permafrost zone.[1] Please refer to Table S1 for specific ecoregion information corresponding to the numbers.

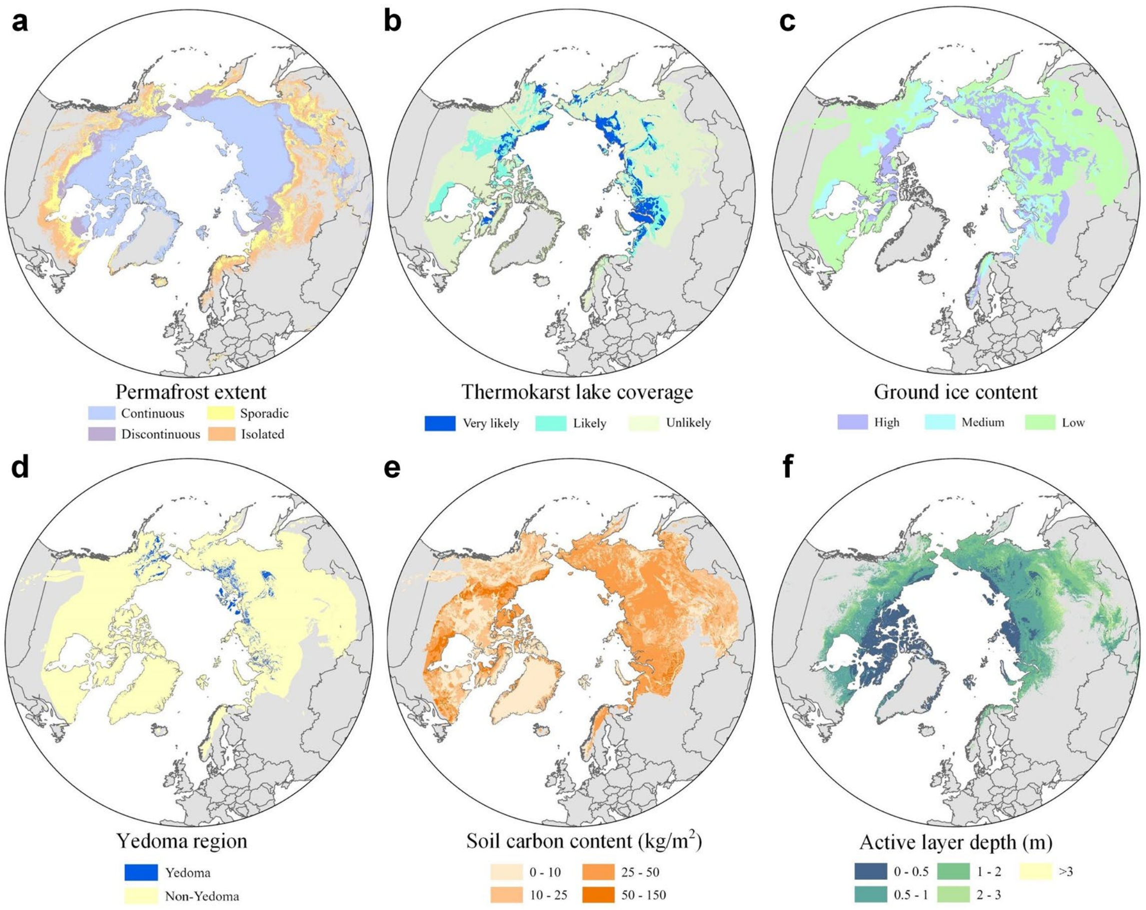

Fig. S5.

Circum-Arctic maps of permafrost-related properties. (a) Permafrost extent[2]: continuous (>90%), discontinuous (50–90%), sporadic (10–50%) and isolated (<10%). (b) Thermokarst lake coverage[3]: very likely (60–100%), likely (1–60%), and unlikely (0–1%). (c) Ground ice content[4]: high (>20%), medium (10–20%), and low (0–10%). (d) Yedoma region[5]. (e) Soil carbon content[6], aggregated from 0–1 m. (f) Active layer depth[7].

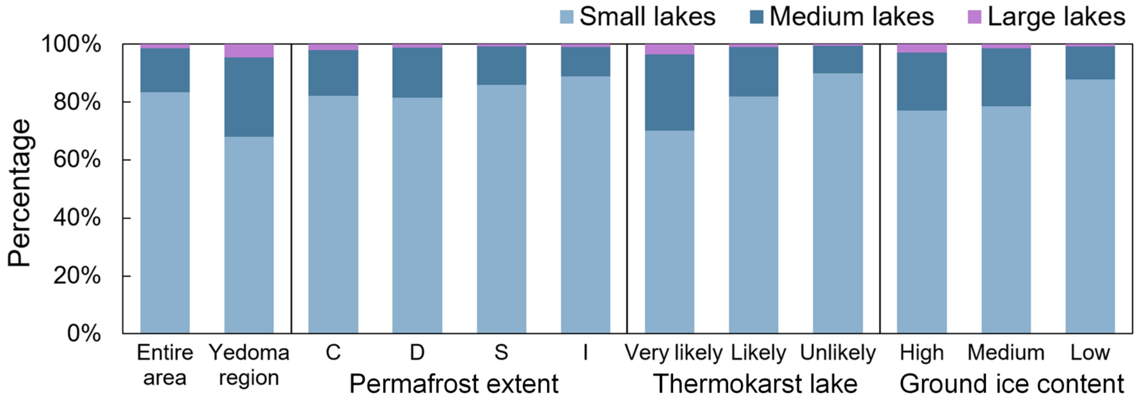

Fig. S6.

Proportion of small, medium, and large drained lakes across various categories of permafrost extent, thermokarst lake likelihood, and ground ice content. C: continuous, D: discontinuous, S: sporadic, I: isolated. Refer to Fig. S5 for classification distribution patterns.

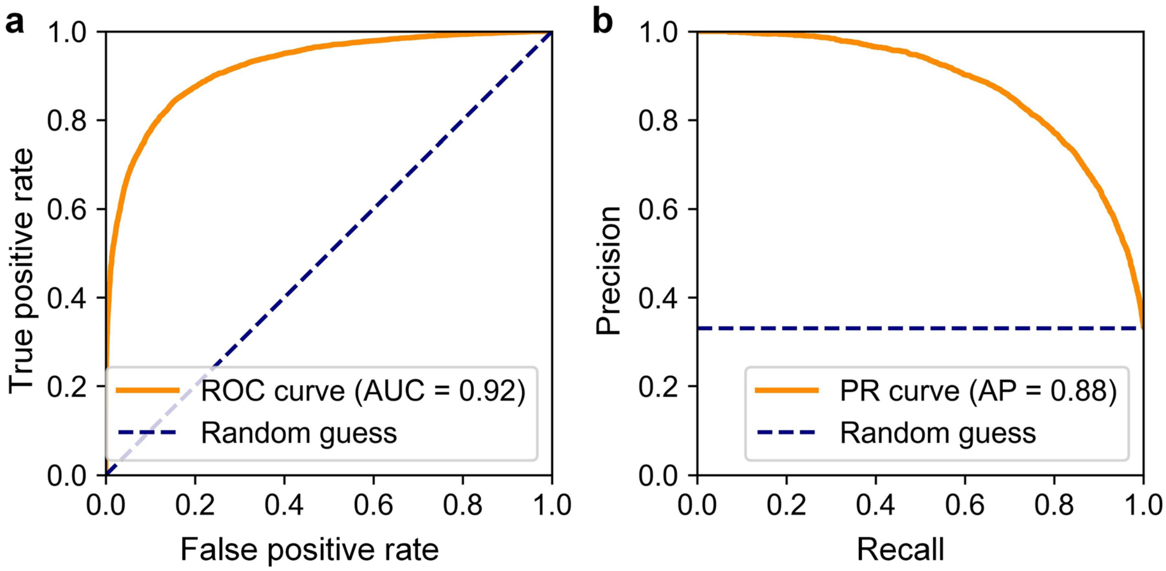

Fig. S7.

Diagnostic assessment of the lake drainage prediction model. (a) Receiver operating characteristic (ROC) curve and (b) precision-recall (PR) curve plots for the accuracy assessment of the machine learning binary classification model used in predicting lake drainage events. The blue dashed lines represent a random guess. Recall: ability to find actual positives; Precision: accuracy of positive predictions; AUC: area under the curve; AP: average precision. A high AUC value indicates that the model has a strong ability to discriminate between positive (drained lakes) and negative (undrained lakes) samples, while a high AP value signifies high predictive quality for positive samples.

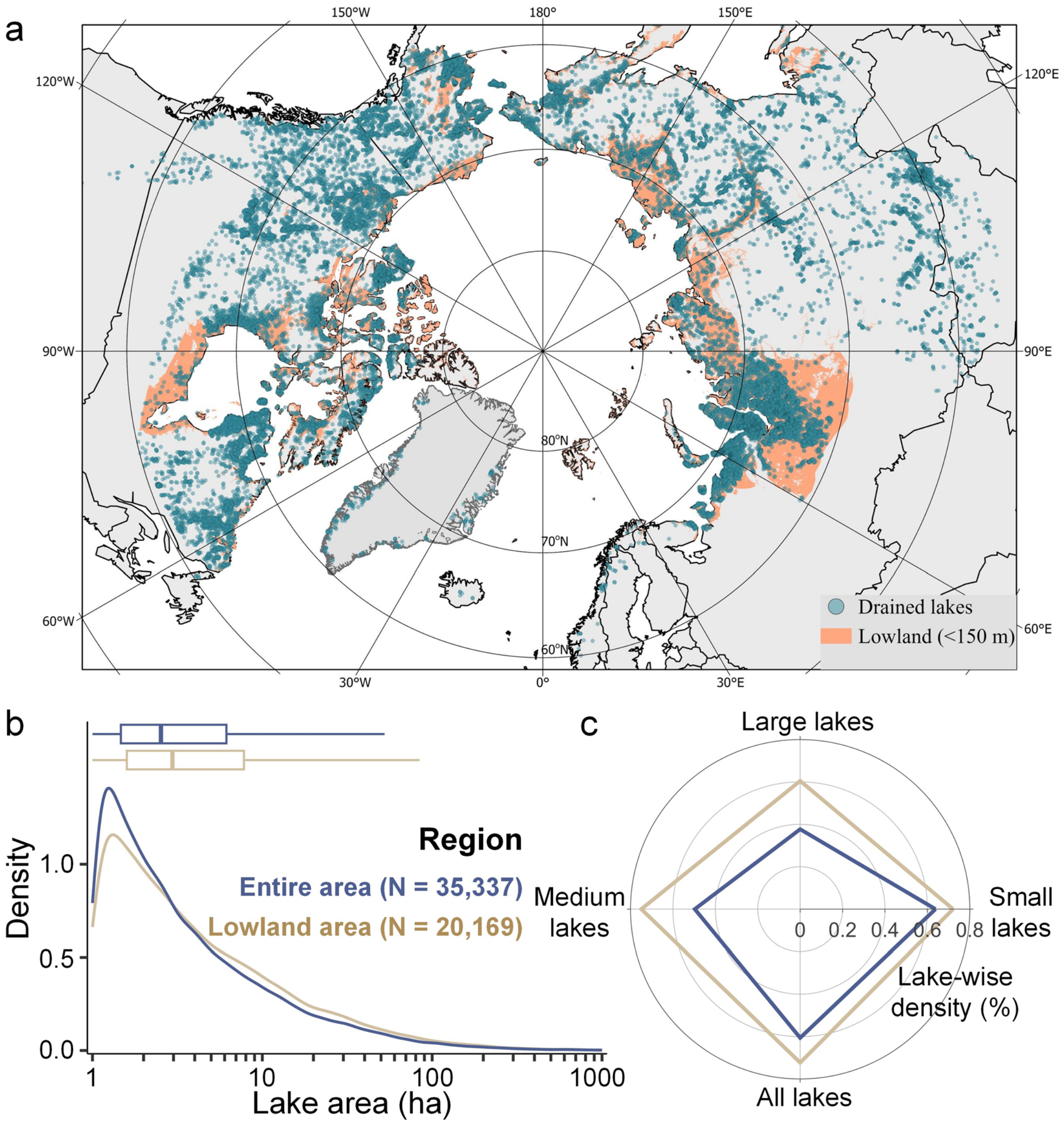

Fig. S8.

Drained lakes in lowland areas. (a) Distribution map of drained lakes, with areas below 150 meters in elevation shown in orange. (b) Area distribution of drained lakes in lowland areas. (c) Lake-wise density for drained lakes of different sizes. Lowlands cover 29.6% of the northern permafrost region, but contribute to 57.1% of drained lakes. Lake-wise density for drained lakes of all sizes in lowland areas exceed the regional average.

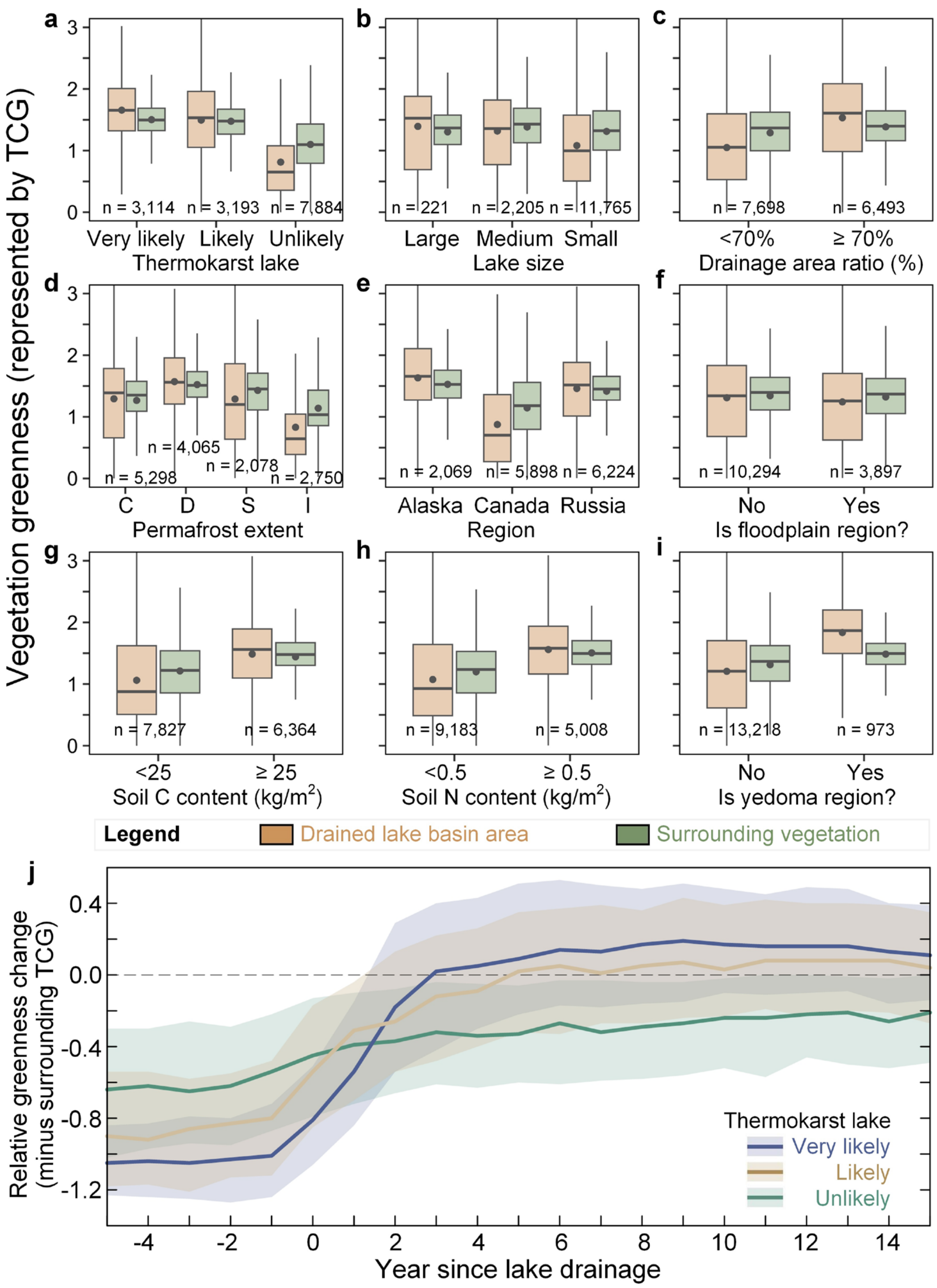

Fig. S9.

Differences in vegetation greenness between DLBs and surrounding areas. TCG measured in the tenth year after lake drainage for various classifications of (a) thermokarst lake likelihoods, (b) lake sizes, (c) drainage ratios, (d) permafrost extents, (e) regions, (f) floodplain status, (g) soil carbon contents, (h) soil nitrogen contents, and (i) Yedoma region. Boxplots show the statistics – horizontal lines: median; dots: mean; boxes: interquartile range; whiskers: 1.5 times the interquartile range. Sample sizes are indicated below each plot. (j) Time series of changes in relative greenness of very likely, likely and unlikely thermokarst DLBs, represented by TCG differences compared to surrounding vegetation. Solid lines show median values, while shaded areas indicate upper and lower quartile ranges.

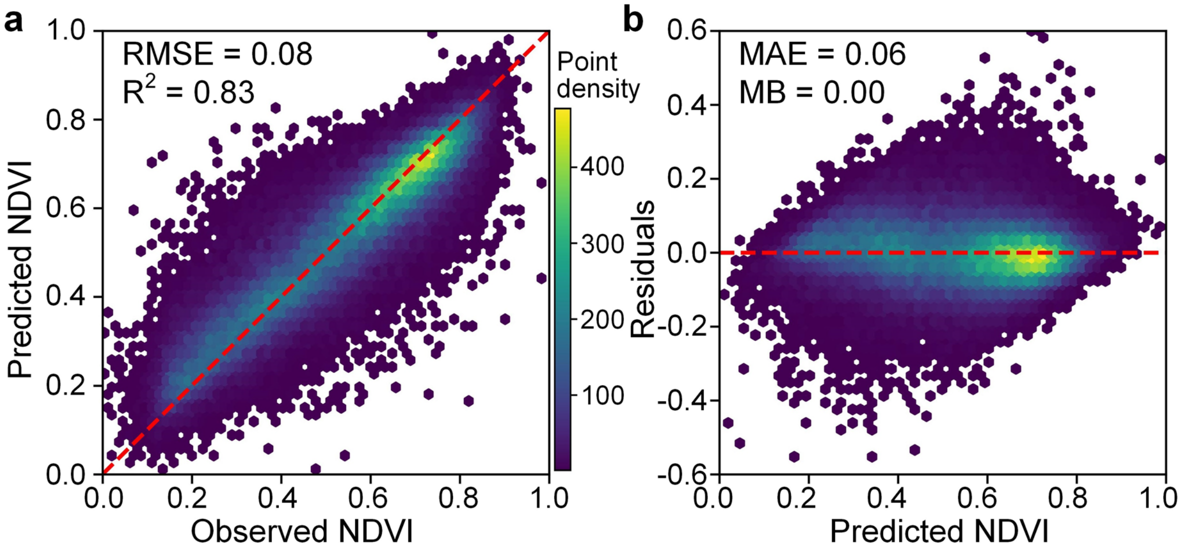

Fig. S10.

Diagnostic assessment of the prediction model for NDVI changes in post-drainage DLBs. (a) Scatter plot comparing predicted and actual values and (b) residual plot of the machine learning regression model for predicting post-drainage NDVI in DLBs. Scatter point density is color-coded, with a red dashed line representing the ideal scenario. RMSE: root mean square error; MAE: mean absolute error; MB: mean bias.

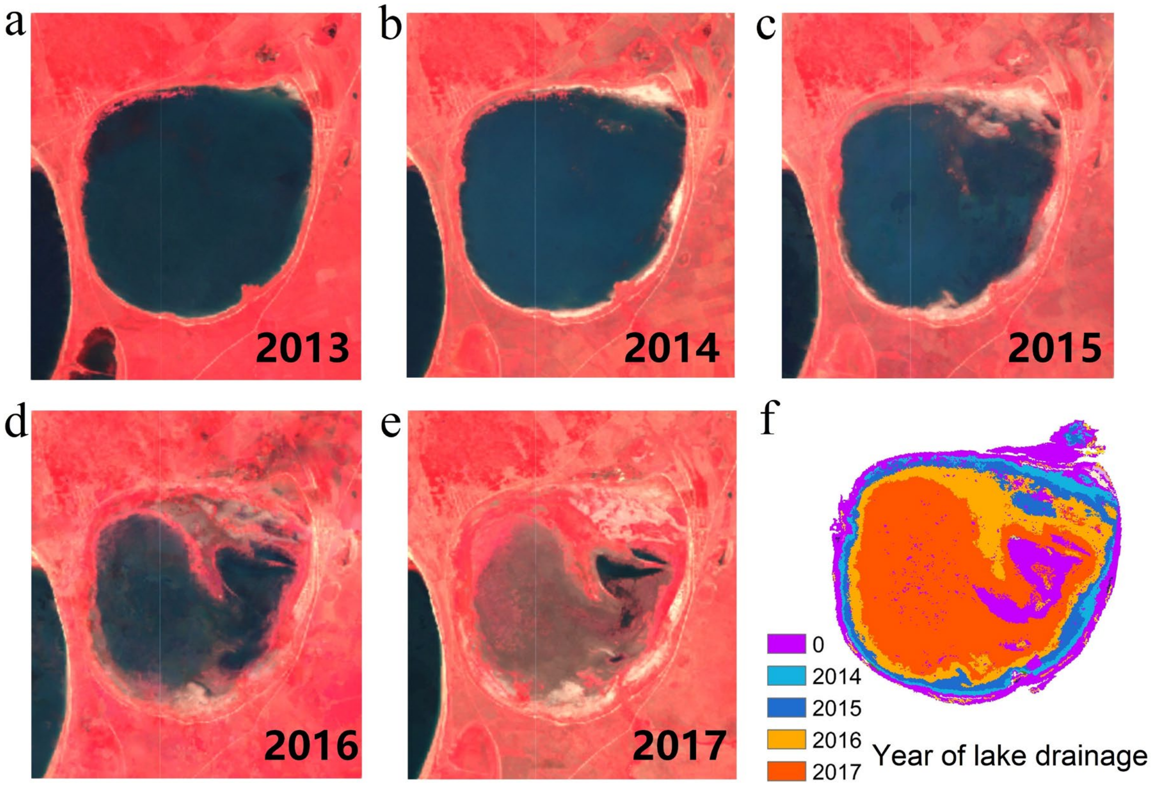

Fig. S11.

Example of gradual drainage of a giant lake. (a–e) Landsat color-infrared images (NIR-R-G) captured at 111°39′ E, 52°40′ N between 2013 and 2017. (f) Year of lake drainage detected by the LandTrendr algorithm on a pixel-by-pixel basis. The lake is Ozero Maloye Yeravnoye Lake, located in the discontinuous permafrost zone in southern Russia, with an area of about 6,000 ha. This shallow lake, with an average depth of only 1.8 meters, experienced drainage between 2014 and 2017. During the winter of 2013, there was an abnormally high amount of snowfall in the lake area, setting a record for the highest snowfall in nearly 20 years. An abnormally thick snowpack would not only hinder the refreezing of the active layer at the lake bottom, leading to talik development and potential drainage channel formation, but would also increase snowmelt, intensifying the possibility of bank overtopping.

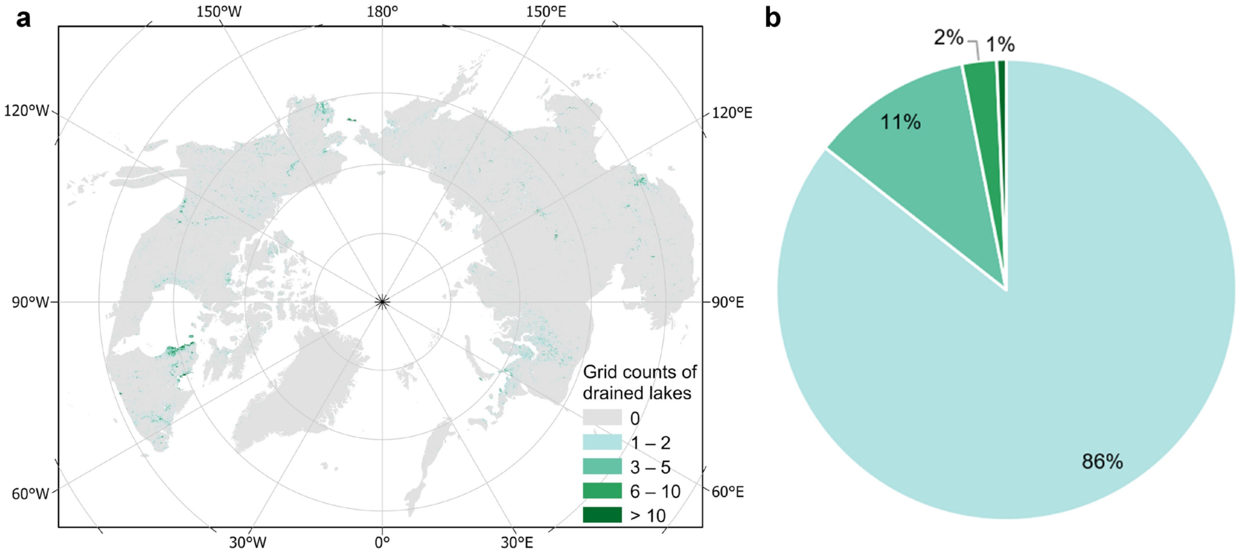

Fig. S12.

Grid-based statistics of drained lakes. (a) Distribution map of drained lakes based on a 0.1°×0.1° grid. (b) Pie chart statistics of grid counts with drained lakes. A total of 35,337 drained lakes are distributed across 20,132 grid cells, with the majority of cells containing 1–2 drained lakes. More than 99% of grid cells have fewer than 10 drained lakes.

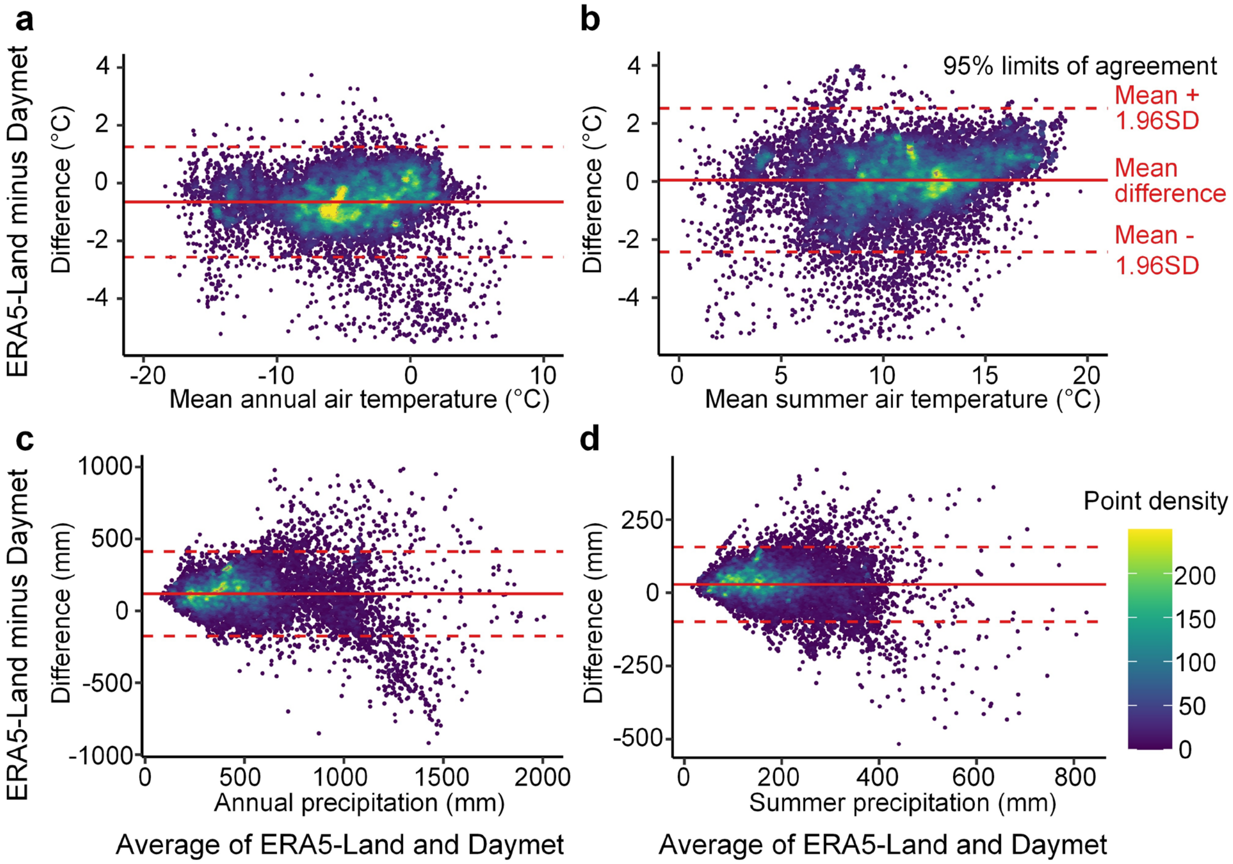

Fig. S13.

Bland-Altman plots for assessing the consistency of ERA5-Land and Daymet reanalysis data. Evaluation of (a) mean annual air temperature, (b) mean summer air temperature, (c) annual precipitation, and (d) summer precipitation. The analysis is based on a total of 16,104 drained lakes detected in North America, using climate data for the year of drainage for comparison. As Daymet dataset does not provide mean air temperature, a simple estimation was made using the average of maximum and minimum air temperature. Bland-Altman plots visually display the average difference and variability between the two datasets. The x-axis represents the average values of the two datasets, and the y-axis shows the differences. The solid red line represents the mean difference, while the dashed red line indicates the 95% limits of agreement. Scatter density is shown in different colours, with yellow representing areas of high concentration of sample points. Overall, ERA5-Land and Daymet reanalysis data exhibit consistency in simulating temperature and precipitation, with relatively low variability.

Table S1.

Basic information about the ecoregions. The locations of the ecoregions are shown in Fig. S4.

No.	Ecoregion name	Region	Latitude range	Longitude range	Area (10^4 km^2)
1	British Columbia mainland coastal forests	Canada	47.5~56.5	-130.3~-120.9	5.3
2	Central Canadian Shield forests	Canada	50.0~55.0	-92.0~-69.9	16.5
3	Copper Plateau taiga	Alaska	61.0~62.9	-147.4~-142.2	1.7
4	Eastern Canadian forests	Canada	48.7~54.6	-73.8~-55.8	19.0
5	Eastern Canadian Shield taiga	Canada	51.4~59.5	-79.8~-55.6	75.6
6	Interior Alaska-Yukon lowland taiga	Alaska/Canada	59.7~68.8	-163.9~-135.2	44.6
7	Mid-Continental Canadian forests	Canada	53.6~61.5	-116.6~-97.9	15.7
8	Midwestern Canadian Shield forests	Canada	52.8~59.3	-111.2~-91.4	39.8
9	Muskwa-Slave Lake forests	Canada	57.0~66.5	-130.0~-113.0	26.4
10	Northern Canadian Shield taiga	Canada	56.6~67.0	-118.9~-94.2	61.7
11	Northern Cordillera forests	Canada	56.4~62.7	-138.0~-122.8	26.4
12	Northwest Territories taiga	Canada	60.2~69.0	-136.3~-114.6	34.8
13	Southern Hudson Bay taiga	Canada	50.4~59.0	-95.8~-75.7	31.7
14	Yukon Interior dry forests	Canada	58.9~63.7	-138.8~-131.3	6.3
15	Canadian Aspen forests and parklands	Canada	50.1~59.0	-122.1~-113.8	1.6
16	Alaska-St. Elias Range tundra	Alaska/Canada	59.2~64.2	-155.3~-136.8	15.0
17	Arctic coastal tundra	Alaska/Canada	68.3~74.2	-163.1~-122.6	9.9
18	Arctic foothills tundra	Alaska/Canada	67.0~70.2	-166.8~-135.7	13.0
19	Beringia lowland tundra	Alaska	57.6~67.1	-173.1~-154.2	13.4
20	Beringia upland tundra	Alaska	58.5~66.4	-171.8~-157.5	9.8
21	Brooks-British Range tundra	Alaska/Canada	65.8~69.7	-163.9~-135.0	16.1
22	Davis Highlands tundra	Canada	65.5~73.7	-80.8~-61.3	8.8
23	High Arctic tundra	Canada	66.6~83.1	-123.1~-61.1	46.6
24	Middle Arctic tundra	Canada	60.2~68.7	-161.7~-130.0	103.7
25	Kalaallit Nunaat low arctic tundra	Greenland	59.8~74.7	-57.7~-32.0	17.0
26	Low Arctic tundra	Canada	55.7~69.8	-132.8~-69.1	80.1
27	Interior Yukon-Alaska alpine tundra	Alaska/Canada	60.2~74.6	-125.6~-63.0	23.4
28	Ogilvie-MacKenzie alpine tundra	Canada	60.7~67.3	-143.3~-125.4	21.0
29	Pacific Coastal Mountain icefields and tundra	Alaska/Canada	54.8~62.0	-151.0~-129.0	7.5
30	Da Hinggan-Dzhagdy Mountains conifer forests	Russia	51.6~54.5	119.7~132.0	9.7
31	Sayan montane conifer forests	Russia	49.6~55.4	86.2~106.7	29.7
32	East Siberian taiga	Russia	51.3~71.4	86.3~142.6	381.3
33	Northeast Siberian taiga	Russia	58.8~70.4	125.0~169.9	113.1
34	Okhotsk-Manchurian taiga	Russia	45.5~58.4	130.8~141.4	31.2
35	Scandinavian and Russian taiga	Scandinavian	59.2~70.0	7.5~62.5	14.3
36	Trans-Baikal conifer forests	Mongolia/Russia	48.1~55.3	103.7~114.6	20.0
37	West Siberian taiga	Russia	59.0~68.3	59.4~90.0	97.3
38	Daurian forest steppe	Mongolia/Russia	47.2~53.2	104.4~120.0	20.7
39	Mongolialian-Manchurian grassland	Mongolia	45.1~51.5	101.9~120.0	18.3
40	Selenge-Orkhon forest steppe	Mongolia	45.8~52.7	92.7~110.8	22.8
41	Bering tundra	Russia	59.8~67.5	156.3~180.0	47.2
42	Cherskii-Kolyma mountain tundra	Russia	59.1~71.7	126.3~170.2	56.0
43	Chukchi Peninsula tundra	Russia	64.3~70.1	162.2~180.0	29.4
44	Kola Peninsula tundra	Russia	66.3~70.9	28.6~41.3	3.1
45	Northeast Siberian coastal tundra	Russia	68.6~72.9	130.3~161.9	22.0
46	Northwest Russian-Novaya Zemlya tundra	Russia	64.1~74.0	42.0~68.2	26.2
47	Novosibirsk Islands arctic desert	Russia	73.2~76.8	135.4~150.9	3.4
48	Scandinavian Montane Birch forest and grasslands	Scandinavian	58.9~71.1	6.3~30.8	16.1
49	Taimyr-Central Siberian tundra	Russia	64.7~77.7	79.2~130.3	94.9
50	Yamal-Gydan tundra	Russia	65.5~73.5	65.3~86.3	40.6

Table S2.

Ecologically-based statistics on lake drainage occurrences and post-drainage vegetation dynamics. NDVI values are derived from the 10th year after drainage. The term "Ratio" refers to the ratio of NDVI in DLBs to the surrounding NDVI.

Ecoregion	Drained lake count				Regional	Drain ratio (%)				NDVI in	Surrounding	Ratio
No.	Small	Medium	Large	All	lake count	Small	Medium	Large	All	DLBs	NDVI	
1	72	2	1	75	4.33E+03	2.05	0.28	1.00	1.73	0.63	0.88	0.71
2	61	2	0	63	5.94E+04	0.15	0.01	0.00	0.11	0.76	0.78	0.98
3	93	9	0	102	5.76E+03	1.93	1.05	0.00	1.77	0.65	0.71	0.91
4	385	13	0	398	7.06E+04	0.72	0.09	0.00	0.56	0.65	0.75	0.87
5	1764	54	0	1818	4.58E+05	0.50	0.06	0.00	0.40	0.55	0.66	0.83
6	887	215	11	1113	7.36E+04	1.46	1.81	1.00	1.51	0.75	0.73	1.02
7	428	69	15	512	2.20E+04	2.45	1.85	1.77	2.33	0.81	0.78	1.03
8	160	3	0	163	1.10E+05	0.24	0.01	0.00	0.15	0.68	0.72	0.95
9	390	24	1	415	3.19E+04	1.53	0.42	0.14	1.30	0.73	0.74	0.99
10	583	77	2	662	4.40E+05	0.18	0.07	0.02	0.15	0.58	0.66	0.88
11	294	13	0	307	2.33E+04	1.50	0.40	0.00	1.32	0.56	0.68	0.83
12	1114	139	2	1255	1.57E+05	0.91	0.45	0.06	0.80	0.64	0.71	0.91
13	233	25	0	258	1.58E+05	0.17	0.13	0.00	0.16	0.59	0.55	1.09
14	66	3	0	69	6.47E+03	1.29	0.26	0.00	1.07	0.67	0.72	0.93
15	205	59	5	269	1.69E+03	13.36	43.07	38.46	15.96	0.69	0.78	0.88
16	272	13	1	286	2.07E+04	1.48	0.62	0.38	1.38	0.29	0.56	0.52
17	130	35	2	167	8.13E+04	0.21	0.20	0.06	0.21	0.39	0.49	0.81
18	66	31	6	103	2.79E+04	0.30	0.60	0.97	0.37	0.72	0.66	1.10
19	1656	366	28	2050	1.19E+05	1.70	1.92	1.28	1.73	0.74	0.64	1.15
20	470	87	2	559	1.89E+04	2.87	3.72	0.97	2.95	0.73	0.65	1.12
21	44	4	0	48	1.29E+04	0.40	0.24	0.00	0.37	0.55	0.60	0.92
22	128	10	2	140	2.21E+04	0.71	0.29	0.28	0.63	0.09	0.12	0.69
23	511	25	1	537	9.93E+04	0.61	0.19	0.05	0.54	0.18	0.21	0.82
24	2227	220	13	2460	1.01E+06	0.27	0.13	0.07	0.24	0.27	0.40	0.69
25	91	11	1	103	3.58E+04	0.35	0.14	0.04	0.29	/	/	/
26	4465	408	13	4886	7.72E+05	0.73	0.28	0.08	0.63	0.37	0.57	0.65
27	256	19	0	275	1.56E+04	1.93	0.90	0.00	1.76	0.39	0.67	0.58
28	202	7	0	209	1.33E+04	1.74	0.47	0.00	1.57	0.40	0.53	0.75
29	200	24	3	227	8.14E+03	2.86	2.42	1.97	2.79	0.30	0.60	0.50
30	77	6	0	83	4.31E+03	1.94	1.93	0.00	1.93	0.55	0.81	0.68
31	115	12	0	127	1.59E+04	0.85	0.56	0.00	0.80	0.54	0.69	0.79
32	1758	366	26	2150	2.54E+05	0.84	0.91	0.77	0.85	0.77	0.78	0.99
33	1191	210	72	1473	1.30E+05	1.27	0.69	1.22	1.13	0.72	0.70	1.04
34	232	5	0	237	1.15E+04	2.31	0.39	0.00	2.06	0.70	0.80	0.87
35	60	8	0	68	3.31E+04	0.22	0.15	0.00	0.21	0.58	0.77	0.75
36	134	53	1	188	8.80E+03	1.72	5.91	0.92	2.14	0.68	0.65	1.05
37	1558	326	21	1905	4.16E+05	0.45	0.55	0.31	0.46	0.66	0.66	1.00
38	312	181	30	523	6.02E+03	6.20	21.14	23.44	8.69	0.40	0.51	0.80
39	242	126	23	391	5.85E+03	5.19	12.03	16.31	6.69	0.30	0.50	0.60
40	77	32	5	114	6.28E+03	1.43	4.05	4.20	1.82	0.31	0.52	0.59
41	515	179	8	702	7.05E+04	0.89	1.52	0.71	1.00	0.78	0.67	1.17
42	175	7	3	185	3.57E+04	0.59	0.13	0.41	0.52	0.42	0.61	0.68
43	290	84	10	384	4.90E+04	0.72	1.08	1.29	0.78	0.75	0.63	1.18
44	63	1	0	64	1.27E+04	0.60	0.05	0.00	0.51	0.62	0.73	0.84
45	115	83	41	239	1.19E+05	0.13	0.32	0.74	0.20	0.70	0.62	1.13
46	1706	371	3	2080	1.27E+05	1.58	2.13	0.23	1.64	0.70	0.67	1.05
47	88	11	1	100	5.86E+03	1.75	1.63	0.64	1.71	/	/	/
48	41	0	1	42	3.78E+04	0.14	0.00	0.09	0.11	0.38	0.58	0.66
49	841	191	15	1047	4.19E+05	0.24	0.28	0.25	0.25	0.63	0.63	1.00
50	1578	1036	114	2728	3.00E+05	0.66	1.79	2.35	0.91	0.69	0.64	1.07

Table S3.

Candidate explanatory variables for the binary prediction model of lake drainage events.

Class	Variable	Source & description
Climate	temperature_2m, snowmelt, snowfall, total_precipitation, total_evaporation, soil_temperature_level_1, wind speed (uv_component), surface_net_solar_radiation	Ref. 8<br>Calculates the annual mean, summer mean, and Sen's slope values
Terrain	elevation, slope	Ref. 9
Permafrost	permafrost extent, Yedoma region, ground ice content, active layer depth, thermokarst lake coverage	Refs. 2–7
Other	latitude, longitude, lake area, ecoregion	Ref. 1

Table S4.

Candidate explanatory variables for the regression prediction model of NDVI in DLBs.

Class	Variable	Source & description
Climate	temperature_2m, snowfall, total_precipitation, total_evaporation, soil_temperature_level_1, volumetric_soil_water_layer_1, surface_net_solar_radiation	Ref. 8<br>Calculates the annual mean, summer mean, and Sen's slope values
Terrain	elevation, slope	Ref. 9
Soil	soil carbon content, soil nitrogen content, ground ice content, active layer depth	Refs. 4,6,7
Permafrost	permafrost extent, Yedoma region, thermokarst lake coverage	Refs. 2,3,5
Other	latitude, longitude, lake area, drainage ratio, year since lake drainage, floodplain, ecoregion	Refs. 1,10

Table S5.
Control parameters of the Landtrendr algorithm.

Parameter	Value	Definition
bestModelProportion	1.0	P-value proportion threshold for fit models with most vertices
maxSegments	4	Maximum number of segments to be fitted on the time series
minObservationsNeeded	6	Min observations needed to perform output fitting
preventOneYearRecovery	True	Prevent segments that represent one-year recoveries
pvalThreshold	0.1	Model with p-value exceeding this threshold will be discarded
recoveryThreshold	0.5	Disallow segments if recovery rate > 1/recoveryThreshold
spikeThreshold	0.8	Spike dampening threshold (1.0 means no dampening)
vertexCountOvershoot	3	Maximum number of vertices that the initial model can exceed

Table S6.
Optimal model hyperparameters for the CatBoost binary classification model used to predict lake drainage events.

Parameter	Value	Description
bagging_temperature	1	Strength of bagging
border_count	255	Splits considered for categorical features
colsample_bylevel	1	Controls feature sampling per level to enhance robustness
depth	10	Max depth of individual trees
early_stopping_rounds	30	Stops training early if validation performance doesn't improve
iterations	1000	Number of boosting iterations (trees)
l2_leaf_reg	1	L2 regularization for leaf weights
learning_rate	0.05	Rate of weight adjustment in boosting
min_data_in_leaf	1	Minimum samples in a leaf node
random_strength	0.6	Amount of noise in tree building
subsample	1	Trains trees on a fraction of data to reduce overfitting

Table S7.
Optimal model hyperparameters for the CatBoost regression model used to predict NDVI in DLBs.

Parameter	Value	Description
bagging_temperature	0.5	Strength of bagging
border_count	128	Splits considered for categorical features
colsample_bylevel	1	Controls feature sampling per level to enhance robustness
depth	10	Max depth of individual trees
early_stopping_rounds	30	Stops training early if validation performance doesn't improve
iterations	2000	Number of boosting iterations (trees)
l2_leaf_reg	1	L2 regularization for leaf weights
learning_rate	0.2	Rate of weight adjustment in boosting
min_data_in_leaf	10	Minimum samples in a leaf node
random_strength	0.8	Amount of noise in tree building
subsample	0.5	Trains trees on a fraction of data to reduce overfitting

Supplementary References:

1. Olson, D. M. et al. Terrestrial Ecoregions of the World: A New Map of Life on Earth: A new global map of terrestrial ecoregions provides an innovative tool for conserving biodiversity. Bioscience 51, (2001).

2. Obu, J. et al. Northern Hemisphere permafrost map based on TTOP modelling for 2000–2016 at 1 km^2 scale. Earth-Science Reviews 193, 299–316 (2019).

3. Olefeldt, D. et al. Circumpolar distribution and carbon storage of thermokarst landscapes. Nat Commun 7, (2016).

4. Brown, J., Ferrians Jr., O. J., Heginbottom, J. A. & Melnikov, E. S. Circum-Arctic Map of Permafrost and Ground Ice Conditions. USGS Numbered Series (1997).

5. Strauss, J. et al. Circum-Arctic Map of the Yedoma Permafrost Domain. Front Earth Sci (Lausanne) 9, (2021).

6. Hugelius, G. et al. A new data set for estimating organic carbon storage to 3 m depth in soils of the northern circumpolar permafrost region. Earth Syst Sci Data 5, (2013).

7. Obu, J. et al. ESA Permafrost Climate Change Initiative (Permafrost_cci): Permafrost active layer thickness for the Northern Hemisphere, v3.0. NERC EDS Centre for Environmental Data Analysis, (2021). https://dx.doi.org/10.5285/67a3f8c8dc914ef99f7f08eb0d997e23

8. Muñoz-Sabater, J. et al. ERA5-Land: A state-of-the-art global reanalysis dataset for land applications. Earth Syst Sci Data 13, (2021).

9. Morin, P. et al. ArcticDEM; A Publically Available, High Resolution Elevation Model of the Arctic. In Proceedings of the Geophysical Research Abstracts; (2016).

10. Tellman, B. et al. Satellite imaging reveals increased proportion of population exposed to floods. Nature 596, (2021).
