# Supplementary material for: Tracking lake drainage events and drained lake basin vegetation dynamics across the Arctic
Source: Nat Commun. 2023 Nov 15;14:7359. doi: 10.1038/s41467-023-43207-0 (PMC10652023; doi:10.1038/s41467-023-43207-0)
Supplement: Supplementary file 3 — Reporting Summary [file 41467_2023_43207_MOESM3_ESM.pdf]

nature portfolio

Corresponding author(s): Yating Chen

Last updated by author(s): Oct 27, 2023

Reporting Summary

Nature Portfolio wishes to improve the reproducibility of the work that we publish. This form provides structure for consistency and transparency in reporting. For further information on Nature Portfolio policies, see our Editorial Policies and the Editorial Policy Checklist.

Statistics

For all statistical analyses, confirm that the following items are present in the figure legend, table legend, main text, or Methods section.

n/a	Confirmed	
☐	☒	The exact sample size (*n*) for each experimental group/condition, given as a discrete number and unit of measurement
☒	☐	A statement on whether measurements were taken from distinct samples or whether the same sample was measured repeatedly
☐	☒	The statistical test(s) used AND whether they are one- or two-sided<br>*Only common tests should be described solely by name; describe more complex techniques in the Methods section.*
☒	☐	A description of all covariates tested
☒	☐	A description of any assumptions or corrections, such as tests of normality and adjustment for multiple comparisons
☐	☒	A full description of the statistical parameters including central tendency (e.g. means) or other basic estimates (e.g. regression coefficient) AND variation (e.g. standard deviation) or associated estimates of uncertainty (e.g. confidence intervals)
☐	☒	For null hypothesis testing, the test statistic (e.g. *F*, *t*, *r*) with confidence intervals, effect sizes, degrees of freedom and *P* value noted<br>*Give P values as exact values whenever suitable.*
☒	☐	For Bayesian analysis, information on the choice of priors and Markov chain Monte Carlo settings
☒	☐	For hierarchical and complex designs, identification of the appropriate level for tests and full reporting of outcomes
☐	☒	Estimates of effect sizes (e.g. Cohen's *d*, Pearson's *r*), indicating how they were calculated

Our web collection on statistics for biologists contains articles on many of the points above.

Software and code

Policy information about availability of computer code

Data collection	Google earth engine, JavaScript scripts are shared in Zenodo https://doi.org/10.5281/zenodo.10043131
Data analysis	python, R codes are shared in Zenodo https://doi.org/10.5281/zenodo.10043131

For manuscripts utilizing custom algorithms or software that are central to the research but not yet described in published literature, software must be made available to editors and reviewers. We strongly encourage code deposition in a community repository (e.g. GitHub). See the Nature Portfolio guidelines for submitting code & software for further information.

Data

Policy information about availability of data

All manuscripts must include a data availability statement. This statement should provide the following information, where applicable:

- Accession codes, unique identifiers, or web links for publicly available datasets
- A description of any restrictions on data availability
- For clinical datasets or third party data, please ensure that the statement adheres to our policy

The drained lakes data generated in this study have been deposited in the Zenodo database under accession code https://zenodo.org/record/7632013#.Y-d7iHZBxD8. The United States Geologic Survey Landsat 5, 7, and 8 Surface Reflectance data used in this study are available from Google Earth Engine at https://developers.google.com/earth-engine/datasets/catalog/LANDSAT_LT05_C02_T1_L2, https://developers.google.com/earth-engine/datasets/catalog/LANDSAT_LE07_C02_T1_L2, https://developers.google.com/earth-engine/datasets/catalog/LANDSAT_LC08_C02_T1_L2. The surface water products published by

the Joint Research Centre (JRC) of the European Commission36 and the Global Land Analysis and Discovery (GLAD) team37 are available at https://global-surface-water.appspot.com/download, https://www.glad.umd.edu/dataset/global-surface-water-dynamics. The global floodplain dataset78 based on MODIS are available at https://developers.google.com/earth-engine/datasets/catalog/GLOBAL_FLOOD_DB_MODIS_EVENTS_V1. The maps of permafrost extent40, lake thermokarst landscape33, Yedoma39, ground ice content41, ecoregion map38 are available at https://doi.pangaea.de/10.1594/PANGAEA.888600, https://dx.doi.org/10.3334/ORNLDAAC/1332, https://maps.awi.de/awimaps/projects/public/?cu=ice_rich_yedoma_permafrost, https://doi.org/10.7265/skbg-kf16, https://www.worldwildlife.org/publications/terrestrial-ecoregions-of-the-world. The storage of soil organic carbon80 is available at http://bolin.su.se/data/ncscd/, and the nitrogen content81 at https://bolin.su.se/data/hugelius-2020. The ECMWF Climate reanalysis ERA5-Land87 Monthly data is available at https://developers.google.com/earth-engine/datasets/catalog/ECMWF_ERA5_LAND_MONTHLY_AGGR.

Research involving human participants, their data, or biological material

Policy information about studies with human participants or human data. See also policy information about sex, gender (identity/presentation), and sexual orientation and race, ethnicity and racism.

Reporting on sex and gender	this information has not been collected.
Reporting on race, ethnicity, or other socially relevant groupings	this information has not been collected.
Population characteristics	this information has not been collected.
Recruitment	this information has not been collected.
Ethics oversight	not applicable

Note that full information on the approval of the study protocol must also be provided in the manuscript.

Field-specific reporting

Please select the one below that is the best fit for your research. If you are not sure, read the appropriate sections before making your selection.

☐ Life sciences ☐ Behavioural & social sciences ☒ Ecological, evolutionary & environmental sciences

For a reference copy of the document with all sections, see nature.com/documents/nr-reporting-summary-flat.pdf

Ecological, evolutionary & environmental sciences study design

All studies must disclose on these points even when the disclosure is negative.

Study description	We employed satellite remote sensing techniques to analyze lake drainage and post-drainage vegetation dynamics in Arctic lakes. We quantified the vegetation growth dynamics in DLBs using the Normalized Difference Vegetation Index (NDVI).
Research sample	We used two surface water products (JRC and GLAD) to help identify drained lakes in the Arctic permafrost zone. A total of 35,337 lake drainage events were detected.
Sampling strategy	In this study, we improved the detection rate by combining water loss pixels from the JRC and GLAD products, and filtered out false lake drainage information by setting the criteria of lake area (>1 ha) and drainage area ratio (>50%). This is the most comprehensive quantification of drained lakes across the Arctic and is thus sufficient to support analysis.
Data collection	The remote sensing image data used in this study were collected and processed through the Google Earth Engine cloud platform. Y.C. and A.L. collected the data products used in the study, and download links are provided in the Data Availability Statement.
Timing and spatial scale	The Landsat image data were acquired over the period 2000-2020, with a spatial extent covering the entire northern permafrost region. The generated image composites have an annual temporal resolution and a spatial resolution of 30 metres.
Data exclusions	We identified lake objects as having undergone a drainage event only if their proportion of surface area loss exceeded 50%, which is a more stringent criterion than that used in previous studies, given focus on investigating drastic alterations in Arctic lake systems.
Reproducibility	All attempts to repeat the experiment were successful.
Randomization	Samples were allocated into groups based on the type of attribute.
Blinding	Blinding is not relevant to our study because the remote sensing images acquired and used for analysis in our study are processed data products. The results of the program run are not relevant to blinding.

Did the study involve field work? ☐ Yes ☒ No

Reporting for specific materials, systems and methods

We require information from authors about some types of materials, experimental systems and methods used in many studies. Here, indicate whether each material, system or method listed is relevant to your study. If you are not sure if a list item applies to your research, read the appropriate section before selecting a response.

Materials & experimental systems

n/a	Involved in the study
☒	☐ Antibodies
☒	☐ Eukaryotic cell lines
☒	☐ Palaeontology and archaeology
☒	☐ Animals and other organisms
☒	☐ Clinical data
☒	☐ Dual use research of concern
☒	☐ Plants

Methods

n/a	Involved in the study
☒	☐ ChIP-seq
☒	☐ Flow cytometry
☒	☐ MRI-based neuroimaging
